# Editorial: Toward an understanding of neurodevelopmental disorders: novel insights from molecular, behavioural and neuroinflammatory mechanisms

**DOI:** 10.3389/fpsyt.2024.1444783

**Published:** 2024-06-24

**Authors:** Luca Pangrazzi, Giovanni Provenzano, Giulia Treccani, Luigi Balasco

**Affiliations:** ^1^ Institute for Biomedical Aging Research, Faculty of Biology, University of Innsbruck, Innsbruck, Austria; ^2^ Department of Cellular, Computational and Integrative Biology CIBIO – University of Trento, Trento, Italy; ^3^ Department of Psychiatry and Psychotherapy, University Medical Center of the Johannes Gutenberg-University Mainz, Mainz, Germany; ^4^ Institute of Anatomy, University Medical Center of the Johannes Gutenberg-University Mainz, Mainz, Germany; ^5^ Center for Mind/Brain Sciences CIMeC – University of Trento, Rovereto, Italy

**Keywords:** neurodevelopmental disorders, psychiatric disorders, neurobiology, inflammation, early-life adversities, autism

Neurodevelopmental disorders (NDDs) encompass conditions characterised by abnormal brain development that impact cognition, communication, behaviour, and movement. These disorders, including autism spectrum disorder (ASD), attention-deficit/hyperactivity disorder (ADHD), and intellectual disabilities, represent a significant public health challenge, affecting up to 3% of children globally. Despite advances in our understanding of these disorders, the lack of specific therapies underscores the need for further research into their aetiology and pathophysiology. Recent studies identified numerous gene variants associated with NDDs, ranging from single-nucleotide variants to copy-number variants. These findings point to a wide array of distinct genes linked to NDDs, highlighting the genetic complexity of these disorders.

However, the origins of many NDDs remain unknown, suggesting that factors beyond genetic variants may play crucial roles. Emerging evidence points to neuroinflammatory mechanisms and environmental factors, such as early life adversities, as significant contributors to the development of NDDs. A multidisciplinary approach integrating molecular, behavioural, and neuroinflammatory studies in human and animal models is essential to understand these aspects. This editorial introduces a collection of original research articles aimed at unravelling the complex mechanisms underlying NDDs and explore potential avenues for novel therapeutic strategies.

The genetic landscape of NDDs is vast and intricate. Recent studies have identified novel gene variants contributing to these disorders, thereby deepening our understanding of their molecular underpinnings. For instance, research on ASD has highlighted the role of voltage-gated potassium (Kv) channels in synaptic function. A case-control study by Liu et al. within this Research Topic examines whether genetic variants in the KCNB1 and KCND2 genes, which encode Kv2.1 and Kv4.2 channels, are associated with the risk and severity of ASD. This research revealed that significant associations exist between KCND2 polymorphisms and both the risk and severity of ASD, highlighting the critical role of synaptic regulation in ASD and suggesting potential targets for therapeutic intervention.

Behavioural studies in NDDs provide crucial insights into the functional consequences of genetic and molecular abnormalities. The dopaminergic system, known for its role in motor and reward-motivated behaviours, has been implicated in ASD. Chhabra et al. conducted a comparative study in mouse models of ASD, including idiopathic and syndromic types, which showed alterations in dopamine receptor densities (Chhabra et al.). These neuroanatomical changes, particularly in D1 and D2 receptor binding in the striatum, might shed light on some behavioural traits observed in ASD and bolster the rationale for using dopaminergic drugs like risperidone and aripiprazole in managing symptoms.

Synaptic dysfunction is a crucial mechanism in elucidating ASD pathophysiology. The excitatory/inhibitory (E/I) hypothesis posits that an imbalance in these synaptic processes contributes to ASD. Nardi et al. explored the ionotropic glutamatergic and GABAergic receptor densities in three different mouse models of ASD (the BTBR strain and Fmr1 and Shank3 mutants) and identified specific alterations in excitatory (α-amino-3-hydroxy-5-methyl-4-isoxazolepropionic acid receptor, AMPA and N-methyl-D-aspartate receptor, NMDA) and inhibitory (γ-aminobutyric acid receptor type A, GABAA) receptor densities across various brain regions (Nardi et al.). These models exhibited an increase in GABAA receptor binding density in the dorsal hippocampus and a decrease in binding density in the cerebellum, suggesting that GABAergic transmission alterations might be a unifying factor in ASD pathogenesis.

Environmental factors, particularly early life adversities, profoundly impact neurodevelopment. Adverse experiences during critical periods of brain development can lead to lasting molecular and behavioural alterations, thereby increasing the risk for NDDs. In this context, animal models have been instrumental in elucidating these effects. Notably, research conducted by Brown et al., utilising mouse models with N-methyl-D-aspartate receptor (NMDAR) hypofunction, has revealed that early disruptions in synaptic signalling can lead to social and anxiety-like behaviours (Brown et al.). These insights highlight the critical need to integrate both genetic and environmental perspectives to comprehensively understand the multifaceted nature of NDD.

Despite significant progress, several challenges still need to be addressed in the study of NDDs. The heterogeneity of these disorders makes it difficult to identify universal biomarkers or treatment strategies. Additionally, the interplay of genetic and environmental factors is complex and still needs to be fully understood. Future research should integrate multidisciplinary approaches, including genomics, neuroimaging, and behavioural studies, to build a comprehensive understanding of NDDs.

This research topic highlights the importance of a comprehensive approach to studying NDDs. By combining insights from molecular, behavioural, and neuroinflammatory research, we can uncover the intricate mechanisms that drive these disorders. Ultimately, this knowledge will pave the way for a deeper understanding of NDDs with possible implications for future therapeutic strategies.

## Author contributions

LP: Writing – review & editing. GP: Writing – review & editing. GT: Writing – review & editing. LB: Writing – review & editing, Writing – original draft.

